# Molecular Action of Lenalidomide in Lymphocytes and Hematologic Malignancies

**DOI:** 10.1155/2012/513702

**Published:** 2012-07-24

**Authors:** Jessica M. McDaniel, Javier Pinilla-Ibarz, P. K. Epling-Burnette

**Affiliations:** ^1^Cancer Biology PhD Program, University of South Florida, Tampa, FL 33620, USA; ^2^Department of Immunology, H. Lee Moffitt Cancer Center and Research Institute, Tampa, FL 33612, USA; ^3^Department of Malignant Hematology, H. Lee Moffitt Cancer Center and Research Institute, Tampa, FL 33612, USA

## Abstract

The immunomodulatory agent, lenalidomide, is a structural analogue of thalidomide approved by the US Food and Drug Administration for the treatment of myelodysplastic syndrome (MDS) and multiple myeloma (MM). This agent is also currently under active investigation for the treatment of chronic lymphocytic leukemia (CLL) and non-Hodgkin's lymphoma (NHL), as well as in drug combinations for some solid tumors and mantle cell lymphoma (MCL). Although treatment with lenalidomide has translated into a significant extension in overall survival in MM and MDS and has superior safety and efficacy relative to thalidomide, the mechanism of action as it relates to immune modulation remains elusive. Based on preclinical models and clinical trials, lenalidomide, as well as other structural thalidomide derivatives, enhances the proliferative and functional capacity of T-lymphocytes and amplifies costimulatory signaling pathways that activate effector responses and suppress inflammation. This paper summarizes our current understanding of T- and natural killer (NK) cell pathways that are modified by lenalidomide in hematopoietic neoplasms to inform future decisions about potential combination therapies.

## 1. Introduction

Lenalidomide (Revlimid, CC-5013) is a second-generation synthetic derivative of glutamic acid and thalidomide analogue with antiangiogenic, antitumorigenic, and immunomodulating activity that was realized due to anecdotal immunomodulatory activity in erythema nodosum leprosum (ENL) [1, 2] and in autoimmune disorders [3–5]. Creation of synthetic modifications to the thalidomide backbone led to the discovery of lenalidomide and pomalidomide with 500-fold greater immunomodulatory potency and safer side effect profile compared to the parent drug [6, 7]. Use of lenalidomide in proliferative neoplasms has recently intensified due to the agent's success in MM and MDS where it acts to alter immune homeostasis and modulate inflammation within the bone marrow microenvironment. Studies in relapsed and refractory B-cell chronic lymphocytic leukemia (B-CLL) as well as non-Hodgkin's lymphoma (NHL) solid malignancies such as central nervous system, ovarian, and renal cell carcinoma demonstrate the potential of this drug in diverse neoplastic processes [8, 9]. While the molecular antitumor mechanism and specificity have been extensively studied in preclinical and clinical settings, the future application and design of effective therapeutic combinations with lenalidomide is dependent on understanding the immunomodulatory mechanism and anti-inflammatory properties in the context of the bone marrow milieu, the microenvironmental interactions, and bioactivity within adaptive and innate immune cells.

## 2. Lenalidomide Augments T-Cell Proliferation and Activation

Immunosurveillance of cancer cells is now a well-established principle thought to contribute not only to the quantity, but also to the quality, or immunogenicity, of a tumor during development [10, 11]. Mechanisms regulating innate and adaptive immune responses are carefully orchestrated to detect and remove infected, transformed, or erratically growing cells within the body. Immune tolerance, induced by changes in the microenvironment and within the tumor cells, contributes to neoplastic expansion. Lenalidomide is able to enhance the proliferative and functional capacity of T cells, which augments immune activity through a variety of mechanisms. Thalidomide was first shown to augment T-cell proliferation and cytokine production in the absence of costimulatory molecules without direct mitogenic activity [12]. Early reports of bone marrow lymphoid aggregates in lenalidomide-responsive MDS patients implicated immune modulation in hematological responses to this agent [13]. When a T-cell encounters cognate tumor antigens presented by antigen presenting cell (APCs), there is an increase in a variety of costimulatory molecules, most importantly CD28, that enables a fully competent signal response by T cells [14]. CD28 binds to B7-1 (CD80) and -2 (CD86) molecules on APCs to generate the appropriate response to antigen stimulation. Absence of CD28-APC interaction (Signal 2) in the presence of T-cell receptor ligation (Signal 1) leads to inactivation or anergy of naïve T cells. Thalidomide, and to a greater extent lenalidomide, induces interleukin-2 (IL-2), interferon-*γ* (IFN-*γ*), and TNF-*α* secretion [12] in the absence of CD28 stimulation, suggesting that the drug somehow activates the costimulatory-dependent signaling cascade initiated by Signal 2 [15]. 

**Figure 1:**
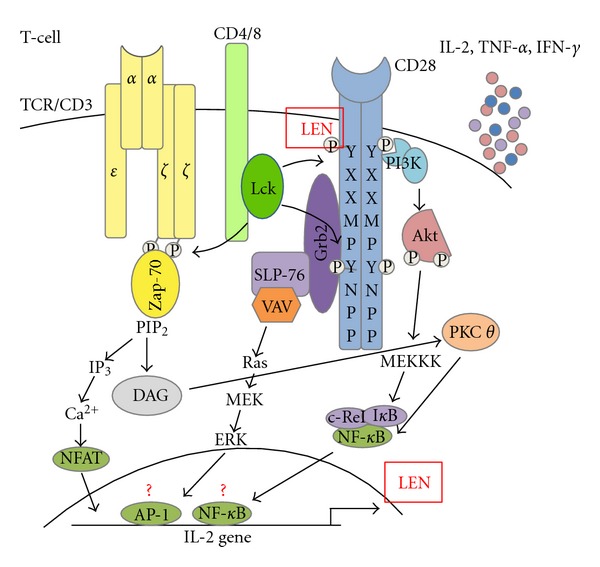
Various T-cell signaling pathways are upregulated after lenalidomide treatment. Lenalidomide is known to have no direct mitogenic activity, therefore it cannot induce proliferation directly. Upon TCR ligation, lenalidomide (LEN) increases phosphorylation of tyrosines within the intracytoplasmic tail of CD28, through an unknown mechanism, increasing downstream signaling and activation of PKC-*θ*, MAPK, and potentially other signaling pathways. These pathways lead to the activation of classic T-cell transcription factors like AP-1, NFAT-1, and NF-*κ*B that induce secretion of the T helper type 1 (Th-1) cytokines interleukin-2 (IL-2), tumor necrosis factor-*α* (TNF-*α*), and interferon-*γ* (IFN-*γ*). Though it is controversial which transcription factors are ultimately increased upon lenalidomide treatment (indicated by a question mark). Upregulation of these pathways potentially reverses T-cell defects, aids in breaking tolerance, and leads to greater CD4+ T-cell help to DCs, NK cells, and CD8+ T cells, augmenting eradication of the tumor cells.

Both Signal 1 (TCR) and Signal 2 (co-stimulation) are necessary for IL-2 production leading to the hypothesis that lenalidomide and the other IMiDs function somewhere within this costimulatory pathway [16–18]. Signaling pathways associated with IL-2 transcriptional activation are shown in Figure 1 and recently reviewed by [19]. The exact differential downstream intermediates emanating from the TCR CD28 are difficult to elucidate because the pathways are integrally connected. LeBlanc et al. showed that lenalidomide acts to increase tyrosine-phosphorylation in the intracellular domain of the CD28 receptor in the absence of costimulatory molecules [20]. Although it is not known if lenalidomide acts directly to induce phosphorylation, the presence of downstream signaling events after treatment such as NF-*κ*B p65 translocation to the nucleus, and cytokine production, suggests that this pathway may be important for lenalidomide's immunomodulatory effect [20]. Others have shown that the activation of PKC-*ζ* and NFAT-2 are important mediators of cytokine production after IMiD treatment [21]. However, a conflicting report showed that PKC-*θ* activity and AP-1 DNA binding was increased, without an increase in NF-*κ*B, OCT-1, and NFAT transcription factor binding, which adds to the controversy about lenalidomide's T-cell-associated molecular mechanism of action [22, 23] (see Figure 1). These controversial results, however, may be attributed to the methods used for T-cell stimulation, namely, TCR stimulation versus calcium channel activation, respectively. Görgün et al. showed that lenalidomide and pomalidomide reduce Suppressor of Cytokine Signaling-1 (SOCS1) expression in T cells, which is an important negative regulator of cytokine signaling [24]. Even when treated with IFN-*γ* to induce SOCS1 expression, the drug was capable of blocking this inhibitory response and potentiating TCR/anti-CD28 costimulation in effector T cells [24]. Although reduction in a suppressive signal may be important, this would not be expected to generate unique responses, such as IL-2, that specifically require a costimulatory signal.

In addition to the activation of effector T cells and NK cells, there is a valid concern about the potential effect of IMiDs on regulatory T (Treg) cells that may deter antitumor immunity by suppressing immunosurveillance [11, 25]. In this regard, lenalidomide and pomalidomide were shown to inhibit the expansion and function of Tregs by downregulating the expression of forkhead box protein 3 (FOXP3) [26, 27]. The preferential augmentation of CD8+ cytotoxic T cells and inhibition of regulatory T cells makes this drug a very interesting and potentially valuable therapeutic candidate to augment immunotherapy responses in cancer patients. 

In addition to the specific effects of lenalidomide on T-cell signaling, our lab and others have shown that the drug alters homeostatic regulation of T cells [28]. In MDS and MM, lenalidomide preferentially acts on specific T-cell memory subsets to reverse immune dysfunction. We found that erythroid responsive MDS patients displayed a greater increase in naïve and central memory T-cell subsets compared to nonresponders. This increase was associated with a concurrent decrease in effector memory subsets, potentially indicating that the drug restores immune homeostasis [28]. A similar increase in central memory T cells was observed by Noonan et al. [29] in MM patients that received lenalidomide in combination with the pneumococcal 7-valent conjugated vaccine (PCV) to establish the principle of vaccine combination therapy. Interestingly, the increase in PCV-specific antibody and cellular responses was specific to the vaccination schedule favoring administration of lenalidomide prior to PCV vaccine. B-CLL, like MDS, is associated with dysfunctional T-cell activity [30, 31] with defects in actin polarization at the immune synapse [32]. Treatment with lenalidomide in CLL restored IL-2 and IFN-*γ* secreting CD4+ and CD8+ T cells to normal levels [33] and reversed the suppressive signals blocking lytic synapse formation [32]. 

Antigen-specific effector T-cell activity *in vitro *and *in vivo *after lenalidomide was demonstrated after treatment for MM, supporting the idea that T-cell reconstitution may be important for antileukemia effects and eradication of myeloma cells [34]. Our studies have shown that lenalidomide is capable of increasing proliferation and cytokine secretion in anergic MDS T cells and indicate that lenalidomide not only improves healthy T-cell function, but also reverses intrinsic cancer-related immune defects associated with deregulated cancer immunosurveillance.

The evidence from *in vitro *and* in vivo *experiments to date, therefore, indicates that lenalidomide has multiple effects on T-cell signaling, but the exact molecular target and mechanism remain elusive. Interestingly, the molecular target mediating thalidomide's teratogenic effects was identified in 2010 by Ito et al. [35]. Using thalidomide-conjugated beads, an E3 ubiquitin ligase, cereblon (CRBN), was shown to directly bind to thalidomide and mediate limb malformation in a zebrafish model. Mutations of two amino acids (Y374A and W376A) in zebrafish CRBN eliminated the drug's ability to interact with the protein and prevented its effects on limb formation. Decreased cereblon expression in MM cells was also recently found to be associated with lenalidomide and pomalidomide resistance [36]. CRBN functions within an E3 complex containing several components including DDB1 and Cullin 4 (Cul4A or Cul4B) that polyubiquitinate (Ub) substrate proteins and mediate their degradation [35]. CRBN and other members of this E3 complex play no known role in T-cell signaling. However, increased Cul4A expression was recently linked to thalidomide response in prostate cancer [37]. Lenalidomide was shown recently by our group to stabilize mouse double minute 2 protein (MDM2) by blocking its autoubiquitination [38]. Since MDM2, like CRBN, is a RING finger E3 ubiquitin ligase, it is possible that IMiDs mediate a class-selective suppressive action against Ub-ligating enzymes, potentially mediating the increase in T-cell signaling.

## 3. Lenalidomide in B-CLL and MM

**Figure 2:**
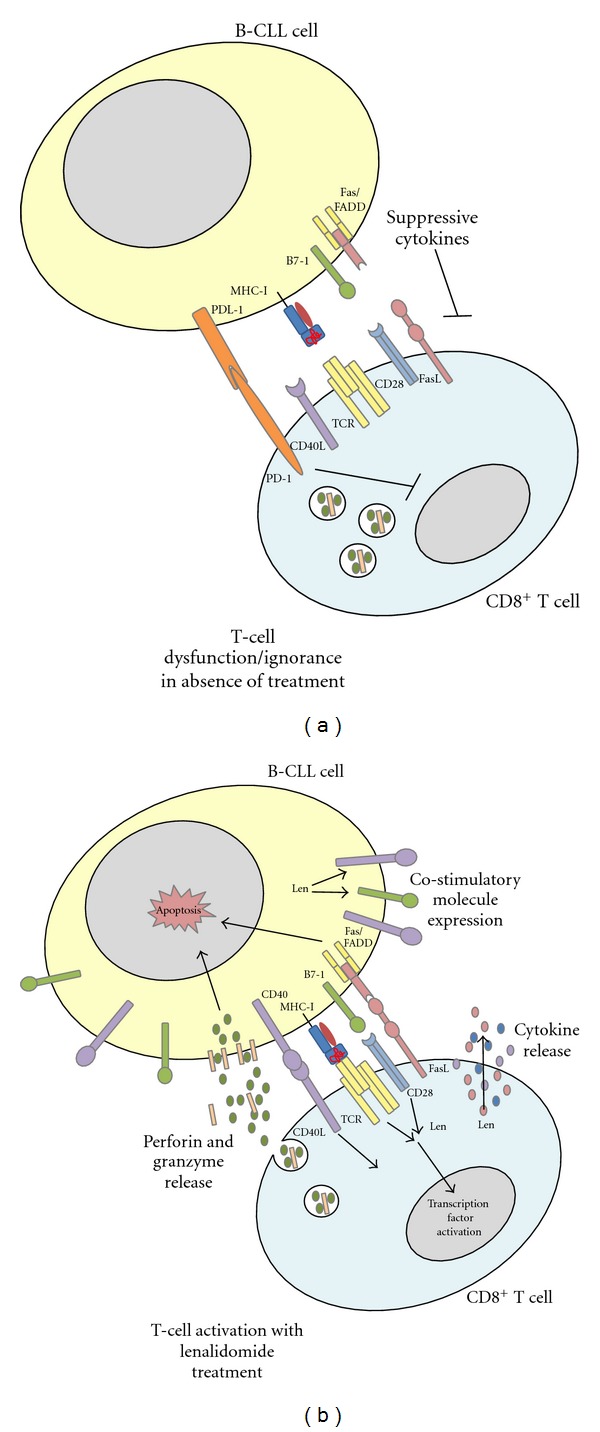
Lenalidomide augments direct CD8+ T-cell killing of B-CLL cells. B-CLL cells are able to evade immune detection through high levels of PDL-1, low levels of costimulatory molecules like B7-1, and a variety of immune-suppressive cytokines in the microenvironment. Lenalidomide treatment (Len) is able to overcome the immune suppression through upregulation of costimulatory molecules like CD40 and B7-1 (CD80/86) on the CLL cells, upregulation of Fas expression, as well as decreasing PDL-1. Through the alteration of surface molecule expression, as well as the increase in T-cell signaling as shown previously, lenalidomide induces better immune synapse formation allowing for increased killing by the CD8+ T cells.

Lenalidomide has proven efficacy in several hematologic malignancies, including MDS, B-CLL, MM, and even some solid tumors attributed to T-cell and NK cell functional reconstitution. B-CLL is the most common leukemia in the United States, and although treatment with nucleoside analog-based chemoimmunotherapies has significantly enhanced outcomes in patients, nearly all of the patients ultimately relapse [39]. Lenalidomide combination treatments for patients with relapsed, refractory, and primary CLL, have resulted in durable hematologic improvement [40–42]. Exposure of primary CLL cells to lenalidomide *in vitro* leads to the induction of costimulatory molecules like CD80, CD86, and FASL on the tumor cells [43], restoring immunological synapse formation and improving autologous tumor cell recognition by T cells [44, 45] (Figure 2). The improved immune synapse formation between T cells and tumor cells was also evident *in vitro* when studied in NHL [46].

The ability of lenalidomide to augment IL-2, IFN-*γ*, and TNF-*α* production from T cells *in vitro* has been described extensively. Similar increases in TNF-*α* production in CLL have been confirmed [47]. Cytokine production and increased T-cell function in CLL is thought to contribute to the tumor flare response (TFR), which is an adverse side effect of lenalidomide treatment that is positively associated with hematologic improvement when properly managed [48]. Since TFR occurs in association with an increase in circulating CD8+ T cells and NK cells, and release of proinflammatory cytokines, it suggests that immunomodulation is important for success of the drug clinically by enhancing the reactivation of immune effector responses against the tumor [47, 48]. Continuous treatment of relapsed refractory CLL patients with lenalidomide was associated with a stable increase in T-cell number in the peripheral blood, which was indicative of a sustained immune response [42]. In B-CLL, both thalidomide and lenalidomide lead to improved tumor recognition. The drug-induced induction of costimulatory molecules on the B-cell tumor cells in CLL resulting in enhanced immune-mediated killing, and decreased tumor burden. 

Impaired differentiation and activation of T and B-cells, as well as NK and dendritic cells, is an important mediator of disease progression in MM [49, 50]. MM is, at present, an incurable B-cell malignancy with abnormal cells accumulating in bone and the bone marrow, which suppress normal hematopoiesis and disrupt the bone marrow microenvironment [51]. Lenalidomide is known in MM to disrupt cellular interactions and adherence of MM to stromal constitutions, decrease growth factors such as IL-6, and induce apoptosis of the neoplastic cells, therefore blocking disease progression [52–54]. The dysregulation of hematopoiesis and increased inflammatory cytokine milieu within the bone marrow microenvironment also contributes to impaired immune effector cell function. Lenalidomide treatment in MM, similar to B-CLL and MDS, reverses T-cell defects directly, but also reverses dendritic cell (DC) dysfunction. DCs from patients with MM have reduced expression, or even absence, of costimulatory molecules [55] and this, along with high levels of IL-6, IL-10, and TGF-*β* within the bone marrow microenvironment, contributes to impaired T-cell costimulation and activation [55, 56]. 

Although an increase in immune activation is associated with drug response and a decrease in tumor burden in CLL, efficacy of the drug has not been definitively shown to be mediated by a direct cytotoxic effect of T cells against the malignant B-cells. Christensen et al. first demonstrated such activity in MM, as lenalidomide treatment in patients *in vivo *increased the killing of *HM1.24+ *myeloma cells by MART-1 specific T cells [34, 57]. Lenalidomide's action on T-cell cytokine secretion, specific tumor cell recognition, and ability to enhance costimulation derived from dendritic cells may all participate in lenalidomide's efficacy for the treatment of MM.

## 4. Immunomodulatory Drugs Increase Natural Killer Cell Recognition and Cytotoxicity of Leukemia Cells

In addition to the potentiating effect on T and B cells, immunomodulatory drugs have a profound effect on the innate immune response, namely, natural killer (NK) cells. NK cells are an important component of the innate immune system where they play major roles in tumor rejection, viral clearance, and DC regulation [58–60]. Thalidomide was shown to enhance the cytotoxic effects of NK cells, as well as increase their cell numbers in MM patients [61]. This enhanced killing effect requires cytokine support from accessory lymphocytes, like T cells, as there is no measurable increase in direct killing of the K562 human leukemia cell line by purified NK cells in the presence of high doses of lenalidomide or pomalidomide [62]. PBMCs depleted of NK cells were not able to kill K562 at all, nor were PBMCs in a transwell experiment, suggesting that NK cells and their contact with the tumor cell is a necessary component of lenalidomide-mediated tumor cell apoptosis [62]. Support from T cells, in the form of IL-2 secretion, is extremely important for NK-cell-mediated cytotoxicity of MM after lenalidomide treatment [21]. Although the combination of lenalidomide with dexamethasone has been shown to have significant activity, IL-2 production was abrogated *in vivo* when MM patients received this combination simultaneously [63]. Hsu et al. demonstrated that dexamethasone treatment suppressed IL-2 production from CD4+ helper T cells, impaired NK cell-mediated cytotoxicity, and countered the immunostimulatory effects of lenalidomide in MM patients. Pharmacodynamic studies may maximize the efficacy of this combination therapy in MM. 

There are multiple mechanisms postulated for increased NK cell killing in the various disease settings. Both pomalidomide and lenalidomide upregulate the expression of CD56, which normally decreases NK killing capacity, but in this setting had no detriment to NK cell killing [62]. Carbone et al. showed that the expression of natural cytotoxic receptors (NCR) and NK receptor member D of the lectin-like receptor family (NKG2D) is necessary for myeloma cell recognition [64] and NKG2D blockade abrogated the effect of lenalidomide in solid tumors [65]. It was recently shown by Benson et al. that the addition of a murine anti-inhibitory killer immunoglobulin receptor (KIR) antibody with concurrent lenalidomide therapy mediated rejection of lenalidomide-resistant tumors in a mouse model [66]. This is similar to their IPH2101 human anti-inhibitory KIR antibody that also increases *in vitro *NK cell cytotoxicity specifically against MM cell targets, but not normal cells, suggesting that clinical testing in combination with lenalidomide is warranted [66]. 

**Figure 3:**
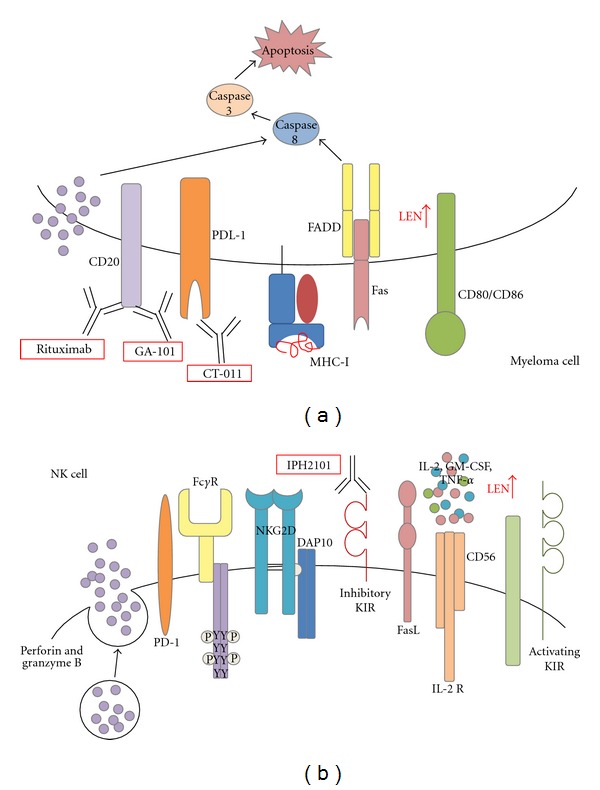
Lenalidomide alone, or in combination with a variety of therapeutic monoclonal antibodies, increases NK-cell-mediated killing of multiple myeloma cells. Lenalidomide (LEN) increases IL-2 secretion from by-standing T helper cells which augments NK cell activity. Lenalidomide, as described previously, upregulates Fas expression and costimulatory molecules on MM cells leading to greater Fas-mediated apoptosis. Lenalidomide has also been shown to augment the ADCC effect of various monoclonal antibodies like Rituximab (anti-CD20), GA-101 (glycoengineered anti-CD20), and CT-011 (anti-PDL-1). CT-011 blocks PD-1 ligand on the MM cells, interfering with binding to PD-1 and inhibiting NK cell activity. Binding of the anti-CD20 antibodies to their targets on MM cells increases complement-dependent cytotoxicity (CDC), as well as NK-cell recognition and killing of the MM cells. IPH2101 is an anti-inhibitory KIR that has been shown in combination with lenalidomide to increase NK-cell killing as well, as blocking the inhibitory signals allows for NK activation and detection of the tumor cells.

A schematic of the various mechanisms of NK cell-mediated killing in MM after lenalidomide treatment in combination with various monoclonal antibodies is shown in Figure 3. MM cells, like most tumor cells, express the programmed death receptor-1 ligand (PD-L1) which downregulates the immune response against malignant cells through programmed death receptor-1 interactions on T cells [67, 68]. Recently, it was shown that NK cells from MM patients express PD-1, and the PD-1/PD-L1 interaction decreased NK cell-mediated killing [69]. A novel anti-PD-1 antibody, CT-011, can increase NK cell-mediated killing of autologous MM cells from patients, without effecting normal cells [69]. This new monoclonal therapy, along with lenalidomide's action of decreasing PD-L1 on MM cells, may improve response rates to this combination therapy.

Enhanced antibody-dependent cytotoxicity (ADCC) by NK cells is also an extremely important mechanism in IMiD function in CLL, MM, and even solid tumors [21, 65, 70]. ADCC is a process where antibodies bind to their ligand antigens on target cells, which then bind to FcR-*γ* receptors on NK cells, and trigger cell lysis through perforin and granzyme-dependent pathways [71]. Lenalidomide- and pomalidomide-induced killing correlates with an increase in Fas ligand (FasL) and granzyme B expression in NK cells, leading to increased ADCC in multiple tumor settings [70]. Thalidomide plus rituximab (RTX), an anti-CD20 monoclonal antibody commonly used in CLL, was found to increase complete response rates in relapsed and refractory MCL patients [72]. Further study of the mechanism showed that the drug-antibody combination increased growth arrest of MCL cell lines, as well as primary cells, compared to RTX alone [73]. Mechanistically, they discovered that lenalidomide enhanced CD20-mAb-dependent apoptosis of the MCL cells by upregulating activation of caspase-3, -8, -9 and the cleavage of PARP, as well as enhanced ADCC by CD16 induction on NK cells [73]. An increase in NK-mediated ADCC is also implicated in the success of RTX and lenalidomide combination therapy in CLL and NHL, although unproven *in vivo* [74, 75]. Ofatumumab, another anti-CD20 monoclonal antibody, binds to a different epitope and induces greater complement-dependent cytotoxicity and has shown evidence of activity in fludarabine and rituximab-refractory CLL [76, 77]. Another CD20 mAb, the glycoengineered GA-101 antibody, induces greater ADCC *in vitro* than RTX and has shown promising preclinical activity in animal models of NHL and B-CLL [78–82]. Lenalidomide therapy is currently being tested with ofatumumab [83] and elotuzumab [84] in advanced, relapsed or refractory patients and has shown therapeutic potential. Therefore, concurrent lenalidomide therapy with these antibodies may prove beneficial in refractory patients to augment antitumorigenic activity through NK cell potentiating effects. 

As an immunomodulatory agent in solid tumors, lenalidomide has been used to reverse tolerance to tumor antigens [85, 86]. As such, lenalidomide may prove beneficial as an adjuvant to vaccine therapies. Wu et al. demonstrated that lenalidomide enhances NK cell killing in a variety of solid tumor cell lines (breast, colorectal cancer, ovary, head and neck, lung cancer, bone sarcoma) treated with cetuximab or trastuzumab [65]. The treatment of hematologic and solid tumors with specific monoclonal antibody therapy concurrently with lenalidomide could potently increase NK cell-mediated tumor lysis and enhance response rates. Lenalidomide induces NK cells to produce granulocyte-macrophage colony-stimulating factor (GM-CSF), TNF-*α*, and various immune recruiting chemokines including RANTES, IL-8, MCP-1, and MIP-1*α*/*β* in response to antibody-coated tumor cell lines, which contributes to a more effective immune response [65]. The IMiDs enhance immunosurveillance in solid and liquid tumor settings through recruiting and activating T and NK cells to suppress malignant growth.

## 5. Summary

This paper summarizes the current information about lenalidomide in proliferative neoplasms and describes our understanding of the molecular mechanism of action in lymphocytes. Based on the overwhelming success of lenalidomide for the treatment of several hematologic malignancies, there is potential for therapies that augment host immune responses to be extended from the relapsed and refractory setting, to primary therapy. Studies over several decades have elucidated the importance of immunosurveillance in malignancy. The seminal discoveries that lenalidomide can potently augment T-cell cytokine secretion and activation in the absence of a secondary signal and augment NK-mediated ADCC in the presence of antibody therapy have only begun to shed light on the mechanism of lenalidomide immune modulating activity. The potential in furthering lenalidomide in combination therapy with therapeutic antibodies, vaccines, and chemotherapy depends on improving our understanding of the molecular mechanism of the drug. 

The mechanism of action and the important molecular and cellular determinants that mediate the immunomodulatory function are poorly understood, yet many cancer patients have benefited from this therapy. T cells and NK cells are rendered anergic or ignorant by the tumor cells through multiple mechanisms related to the lack of costimulation and immunosuppressive signals within the tumor microenvironment. Because of the importance of costimulation in determining the immune response, therapeutic manipulation with lenalidomide has generated particular interest. The mechanism of action is clearly linked to changes in the bone marrow microenvironment, cytokine secretion, regulation of angiogenesis and host antitumor immunity. Since this agent has significant activity in MM, MDS, CLL, NHL, and MCL, a better understanding of the leukemia biology and the molecular targets that mediate the immunomodulatory activity is needed to harness the full potential of this agent in combination therapies.
